# Gaps between Evidence and Practice in Postoperative Radiotherapy for Prostate Cancer: Focus on Toxicities and the Effects on Health-Related Quality of Life

**DOI:** 10.3389/fonc.2016.00070

**Published:** 2016-03-24

**Authors:** Hamid Raziee, Alejandro Berlin

**Affiliations:** ^1^Department of Radiation Oncology, University of Toronto, Toronto, ON, Canada; ^2^Radiation Medicine Program, Princess Margaret Cancer Centre, University Health Network, Toronto, ON, Canada

**Keywords:** prostate cancer, adjuvant, salvage, radiotherapy, quality of life, toxicities

## Abstract

Adjuvant radiotherapy (ART) after prostatectomy for patients with high-risk features [extracapsular extension (ECE), seminal vesicle invasion (SVI), and positive margin] has been shown to be associated with improved biochemical disease-free survival in three large randomized trials and with improved overall survival in one. Similarly, salvage radiotherapy (SRT) can effectively achieve biochemical control in a significant proportion of patients with a rising PSA after surgery. Nonetheless, both approaches of postoperative RT remain highly underutilized. This might be partly due to concerns with overtreatment inherent to adjuvant approaches, and/or hesitance about causing radiation toxicities and their subsequent effects on the patient’s quality of life. Herein, we review the literature lending evidence to these arguments. We show recent series of ART/SRT and their low rates of acute and long-term toxicities, translating only in transient decline in quality-of-life (QoL) outcomes. We conclude that concerns with side effects should not preclude the recommendation of an effective and curative-intent therapy for men with prostate cancer initially treated with radical surgery.

## Introduction: Role of Adjuvant and Salvage Radiotherapy after Prostatectomy and the Underutilization Problem

There were approximately 220,800 new cases of prostate cancer (PCa) diagnosed only in the US in 2015, with 27,540 patients dying from the disease (1). According to a recent analysis based on SEER data, 90% of prostate cancer cases in the US are diagnosed in localized stages, and 40% of these are treated with radical prostatectomy (2). After surgery alone, 30–40% of patients will experience biochemical failure (3–5), and one-third of recurrent cases will be subsequently diagnosed with metastatic disease (6). Nonetheless, death from prostate cancer remains infrequent, and cancer-specific survival rates are above 90% after 15 years of surgery alone (5).

In order to decrease the risk of biochemical failure, particularly in patients with high-risk features (including positive surgical margins, high grade disease, and/or pT3-stage) (7), postoperative radiotherapy has been studied and shown efficacious. Three randomized trials from cooperative groups (SWOG-8794, EORTC 22911, and ARO 96-02) have demonstrated significant biochemical disease-free survival improvement with adjuvant radiotherapy for patients with high-risk features (8–11). Moreover, one of these trials showed superior overall survival in the radiation arm (11). Given these benefits, adjuvant radiotherapy in high-risk patients has been endorsed and recommended by practice guidelines from leading European and North American societies (12–14).

In patients presenting with biochemical recurrence (rising PSA) after prostatectomy, salvage radiation has been reported to achieve an overall biochemical response rate of 50%, translating into a threefold increase in prostate cancer specific survival (15). However, long-term disease control rates are highly variable, ranging from 10 to 40%, mostly due to the intrinsic patients’ heterogeneity in this high-risk population. To date, there is lack of robust predictive markers to identify those with PSA increase due to local recurrence (who are likely to benefit from salvage radiation) from those with already microscopic distant spread (in whom further local therapies is likely futile) (16, 17). At present, no prospective study has directly compared ART vs. SRT approaches. Although such efforts are currently underway, the optimal postoperative RT timing conundrum remains a topic of controversy (18).

Despite the demonstrated benefits of both adjuvant and salvage radiotherapy, these treatments remain strikingly underutilized, with <15% of eligible patients with high-risk features receiving radiotherapy across different jurisdictions (19–25). Moreover, during the last decade, the absolute utilization rates have not significantly changed despite the publication of the three large ART randomized trials (21, 24, 26), notwithstanding the fact that recommendation for the use of adjuvant radiation has increased (25). This discrepancy between evidence and practice is more pronounced in older patients, plausibly due to the uncertainty about treatment benefits in the context of a shorter life span and/or higher comorbidities (21).

To explain this underutilization, some plausible reasons have been suggested in relation to the pivotal trials’ design and outcomes. Related to design, the comparison of ART with observation instead of early SRT (19), not ascertaining the use or timing of SRT in the observation arm, and the inclusion of patients with detectable PSA pre-ART (27) have been mainly discussed. Regarding outcomes, particularly the absence of survival benefit in two of the trials has been highlighted, with improvements shown only in SWOG study, which could have been confounded by comorbidities in the control group (28). Additionally, physician’s specialty appears also to influence ART/SRT use, as demonstrated by urologists being less likely to recommend it compared to radiation oncologists (29, 30). Patient factors, such as age, comorbidities, and life expectancy estimates, have also been suggested to influence endorsement of post-prostatectomy radiation (21).

However, current literature has mostly focused on two major reasons for withholding or deferring the use of postoperative radiotherapy, namely, concerns with overtreatment and radiation toxicities with their subsequent impact on patient’s quality of life. To better understand the delay in practice change, herein, we summarize the literature focusing on these two potential factors. The evidence presented here could also serve to guide treatment individualization and shared decision-making between physicians and patients regarding curative-intent adjuvant and salvage radiotherapy after radical prostatectomy.

### Avoiding Overtreatment or Favoring Undertreatment? Nuances until Superiority (or Non-Inferiority) of SRT Is Proven

Although the bulk of evidence supports the use of immediate postoperative radiation, its proper timing is a matter of debate (28) mainly due to the concerns related to the possibility of overtreatment with early adjuvant radiation. A considerable proportion of high-risk patients achieve good disease control with surgery alone, with slightly over half of them remaining free from biochemical failure at 5 years (10, 31). In patients with adverse pathological features, such as ECE, positive margins, and SVI, the 10-year progression-free probability can be as high as 71, 44, and 37%, respectively (32, 33). Therefore, the alternative concept of delaying radiotherapy to the time of recurrence (i.e., rising PSA) has been proposed by some as an effective method to provide the same results while avoiding the intrinsic overtreatment risk of adjuvant approaches (16).

This treatment strategy, at present time, is supported by retrospective evidence (34), and as the core, assumes that SRT or delayed ART could be as effective as immediate ART (26). Indeed, a pooled analysis of 10 SRT studies has yielded bRFR rates similar to historic reports of adjuvant radiation (71 vs. 67–74%, respectively) (34). However, this indirect comparison in the absence of randomized prospective data cannot safely answer whether early salvage is really equivalent to adjuvant radiation. In fact, matched group analyses have shown superiority of adjuvant over salvage radiotherapy with regards to freedom from biochemical failure (35–37). Solving this clinical conundrum is the objective of ongoing phase III trials, including RADICALS (34), RAVES (35), and GETUG-17 (http://ClinicalTrials.gov identifier NCT00667069), from which informative results will likely be available in the upcoming decade.

Although SRT approaches might be inferior to ART in general, within the former, earlier rather than delayed salvage has shown superior outcomes. The aforementioned pooled analysis on retrospective studies demonstrates improved 5-year biochemical relapse-free survival with early salvage compared to delayed salvage radiation, with improved outcomes in those patients with a PSA level of <0.5 ng/ml. Other studies have suggested different threshold values (34, 38, 39). Acknowledging that within SRT approach, earlier salvage renders more favorable results, the comparison with adjuvant radiation remains unclear given the lack of prospective studies. In addition, clinically applicable and validated PSA thresholds have been hard to determine. The available cutoff points below which SRT is assumed to be equal to ART mostly represent study-specific statistical considerations and might not be used to guide clinical practice until properly validated in a prospective fashion.

Intention to avoid potential overtreatment inherent to adjuvant approaches is a longing that is not exclusive to prostate cancer (40), and one of the principles of personalized cancer treatments is to tailor management to each patient’s disease and individual unique characteristics. When robust and consistent evidence supports the use of adjuvant treatment, the goal for avoiding overtreatment should be to precisely identify those patients in whom the treatment is futile, without precluding *a priori* a significant proportion of patients to derive benefit from such therapy. This requires prospective studies with sufficient follow-up (41), which at present are lacking in postoperative prostate cancer setting. A similar scenario was experienced in determining the role of axillary node dissection in breast cancer patients with positive sentinel node biopsy. Almost 7000 patients were randomized in three separate trials [IBCSG 23-01 (42), AOCSOG Z0011 (43), and EORTC 10981-22023-AMAROS (44)] before a conclusion could be reached regarding the subset of patients where elimination of axillary dissection is safely warranted.

Even if justified, favoring delayed over adjuvant radiotherapy does not seem sufficient to explain the overall low utilization of radiotherapy in post-prostatectomy setting. In a recent US nation-wide practice analysis, the use of immediate (ART) and delayed (SRT) was relatively stable over time, with only a slight increase in delayed RT between 2007 and 2009 (24). Contrary to this, another study has reported a minimal shift toward earlier radiation after the publication of the ART randomized studies (23). These findings together challenge the assertion that the underutilization of ART is due to increased use of SRT, and it seems safe to state that neither immediate nor delayed radiation has been increasingly used despite large trials demonstrating benefits. In current practice, some patients are being precluded of a potentially curable treatment for PCa after initial radical prostatectomy.

### Concerns with Radiation Toxicities: How Much More Evidence Is Needed?

Radiation toxicities and their impact on the quality of life (20, 45) might be another deterrent for the use of ART/SRT. This, in part, can be explained by EORTC and SWOG trials’ reports of increased incidence of late toxicities in the adjuvant RT arm (8, 9). In EORTC trial, grade 2 or higher late GU toxicity was significantly higher in radiation arm (21.3 vs. 13.5%), but late grade 2 GI toxicity rates were similar. Nonetheless, more clinically relevant grade 3 side effects were not significantly different between the two arms (2.5–5%), and no grade 4 events were reported (8). Although the SWOG trial did not report graded toxicity, complications were generally more frequent in the radiation arm (23.8 vs. 11.9%), mainly due to rectal complications (3.3 vs. 0%) and urethral strictures (17.8 vs. 9.5%) (9). Although ART seems to double the relative risk of complications as compared to observation, the absolute rates of long-term toxicities remain low, particularly for high grade side effects. From a benefit/risk analysis based on these early studies, the NNT for improving biochemical relapse rates (1.6 at 10 years) remains significantly better than the NNH (5 for grade 2 or higher and 20 for grade 3 or higher) to present any toxicity during 10-year follow-up.

Both EORTC and SWOG trials used conventional two-dimensional radiotherapy planning (e.g., four-field box), which does not represent state-of-the-art radiation oncology practice. Over the last decade, various and significant technological innovations have been realized in radiation planning and delivery (46). The advent of high-precision radiotherapy has positively impacted the delivery of lower doses to surrounding normal tissues and the subsequent risk of toxicities. With intensity-modulated radiotherapy (IMRT), even more conformal planning is feasible compared to three-dimensional radiotherapy (3DCRT), translating in improved early GI/GU (47) and late GI toxicity profiles (48). Moreover, daily image guidance added to IMRT planning for accurate delivery allows prioritizing rectal dose constraints over target volume coverage. When tested in a recent phase II trial, this technique translated in excellent biochemical control without grade 3–4 acute or late toxicities (45). Although longer follow-up is warranted, the implementation of modern radiotherapy techniques in the post-prostatectomy setting will likely reflect in declining rates of long-term toxicities and subsequent QoL impact, as have indeed been observed in other PCa radiotherapy scenarios (49). Whereas no randomized study has directly compared the toxicities of conventional vs. high-precision planning (and it is unlikely to be conducted), Table 1 summarizes and contrasts the results of benchmark randomized trials and contemporary studies employing state-of-the-art radiotherapy techniques, reporting the toxicity profile of postoperative radiotherapy. Despite variations among groups in the definition of target volumes, doses, and radiation techniques, a very low rate of high grade acute or chronic toxicities is consistent across studies. The majority of adverse events are grade 2, and none of the available reports have described grade 4 toxicities.

**Table 1:** **Summary of post-prostatectomy radiotherapy studies, outcomes, and toxicities**.

Study	Prospective	Number of patients, median follow-up	Technique, total dose	bRFR	Acute G ≥ 2 toxicity (%)		Acute G3–4 toxicity (%)		Late G ≥ 2 toxicity (%)		Late G3–4 toxicity (%)		Change in ED (%)
					GU	GI	GU	GI	GU	GI	GU	GI	
Thompson et al. (9)/Moinpour et al. (50)	Yes (RCT)	425, 10.6 years	Conventional, 60–64 Gy	65.1% (ART) vs. 36% (Obs) (10 years)	NR	NR	NR	NR	24.3^a^	3.3^a^	NR	NR	NR
Bolla et al. (8)	Yes (RCT)	1005, 10.6 years	Conventional, 60 Gy	60.6% (ART) vs. 41.1%(Obs) (10 years)	NR	NR	NR	NR	21.3	2.5	5.3		NR
Wiegel et al. (10)	Yes (RCT)	385, 53.7 months	Conventional, 60 Gy	72% (ART) vs. 54% (Obs) (5 years)	NR	NR	NR	NR	2	1.4	0.5	0	NR
Choo/Pearse et al. (51, 52)	Yes	75, 45.1 months	3DCRT, 66 Gy	78.6% (7 years)	12	18	3	3	22.6	8.7	2.8	1.6	NR
Eldredge et al. (53)	No	68, 15 months	IG-3DCRT, 68.4 Gy	93% (3 years)	15	13	2	0	13.6	5.4	0	3	NR
De Meerleer et al. (54)	No	135, 9 months	IMRT, 74 Gy	67% (3 years)	28	15	3	0	33.8	16	3	3	NR
Ost et al. (55)	No	104, 36 months	IMRT, 74 Gy	93% (3 years)	34.6	22	8	0	26	7	4	0	NR
Goenka et al. (48)	No	176, 53 months	3DCRT, IMRT, ≥70 Gy	39.9% (5 years)	16.3	9.8	NR	0	17	5.2	6	1.4	27
Shelan et al. (56)	No	76, 52 months	IMRT, 70 Gy	62.5% (4 years)	NR	NR	NR	NR	NR	NR	4	0	NR
Wong et al. (57)	No	50, 18.9 months	IG-IMRT, 65 Gy	72.9% (2 years)	8	2	0	0	4	4	0	0	NR
Sandhu et al. (58)	Yes	26, NR	IG-IMRT, 68 Gy	NR	12	4	0	0	NR	NR	NR	NR	NR
Cheng et al. (59)	No	70, 10.6 months	IG-IMRT, 68.8 Gy	NR	36	41	0	0	NR	NR	NR	NR	NR
Nath et al. (47)	No	50, 24 months	IG-IMRT, 68 Gy	NR	14	8	0	0	16	2	2	0	NR
Deville et al. (60)	No	67, 25.5 months	IG-IMRT, 70.2 Gy	NR	16	46	3	0	24	1.5	9	0	NR
Hunter et al. (61)	No	104, 33 months	IG-IMRT, 70 Gy	NR	NR	NR	NR	NR	11.6	0	5.4	0	NR
Chua et al. (62)	Yes	75, NR	IG-IMRT, 66 Gy	NR	30.6	22.6	4	1	NR	NR	NR	NR	NR
Cremers et al. (63)	No	197, 40 months	3DCRT, 63 and 58.5 Gy (2.25 Gy/fr)	59% (5 years)	NR	NR	NR	NR	29.4	1.5	6	0.6	NR
Cortes-Gonzalez et al. (64)	No	184, 48 months	3DCRT, 70 Gy	63% (4 years)	NR	NR	3	0	NR	NR	9	5	NR
Corbin et al. (65)	Yes	78, 24 months	IMRT, 66.6 Gy	NR	NR	NR	NR	NR	NR	NR	NR	NR	NS
van Gysen et al. (66)	Yes	64, 24 months	IMRT, 66 Gy	NR	NR	NR	NR	NR	NR	NR	NR	NR	NS
Berlin et al. (45)	Yes	68, 71.2 months	IG-IMRT, 66 Gy	72.7% (5 years)	38.2	22	0	0	10.6	12.3	0	0	NS

*bRFR, biochemical relapse-free rate; G, grade; G3–4, grade 3–4; GU, genitourinary; GI, gastrointestinal; ED, erectile dysfunction; RCT, randomized controlled trial; 3DCRT, three-dimensional conformal radiation therapy; IG-3DCRT, image-guided three-dimensional conformal radiation therapy; IMRT, intensity-modulated radiation therapy; ART, adjuvant radiotherapy; Obs, observation arm; NR, not reported; NS, non-significant*.

*^a^Toxicity grade not reported*.

There is very limited literature on the quality of life (QoL) and/or patient-reported outcomes after postoperative radiotherapy (Table 2). Moreover, the methodology for measuring and reporting QoL is not uniform, which further limits drawing definite conclusions. After radiation, the available longitudinal data show a transient decline in GI and GU QoL indicators, particularly during the first months. With longer follow-up (e.g., 3–12 months after ART/SRT), QoL metrics return to patient’s pre-radiation baseline or become comparable to reference values in GI, GU, and sexual domains. However, among the studies quantifying long-term symptoms and their impact on QoL, the results are not fully consistent. In the study by Moinpour et al. (50) reporting QoL of SWOG trial’s participants, bowel tenderness and urgency were significantly higher in radiation arm (47 vs. 5% at 6 weeks); however, this negative impact of ART was transient, and no difference between treatment arms was present after 2 years. Pinkawa et al. also report higher rates of bowel bother at a follow-up longer than 12 months, although the mean decrease in score is 4 points compared to baseline (90 vs. 94) (67). This long-term detrimental impact in GI-related QoL has not been observed in other studies. The SWOG quality-of-life analysis also reported long-term impact on urinary frequency subscale (50), where patients reported 15% more frequent urination over the follow-up duration (5 years). Again, this effect trend has not been observed in other reports. Overall differences between these two earlier and the most recent studies could in part be explained by the fact that the former correspond to the pre-IMRT and image-guidance era.

**Table 2:** **Summary of studies on patient-reported QoL indicators**.

Study	Setting	Technique	QoL tool	Urinary domain, mean	Bowel domain, mean	Sexual domain, mean	Global health domain, mean	Comparator^a^	Difference from comparator	Last reported measurement (months)
Moinpour et al. (50)	ART vs. RP only	3DCRT	SWOG QoL Questionnaire	NA	NA	NA	NA	Baseline	SS in favor of control arm: urinary frequency at 60 months, bowel function until 24 months, global QoL at 6 weeks SS in favor of radiation arm: global QoL at 60 months NS at other time points	60
Pinkawa et al. (67)	SRT/ART	4F Box	EPIC	Function, 84 Incontinence, 74	Function, 91 Bother, 90	Function, 11	NR	Baseline	SS in favor of control arm: urinary function at 6 weeks, bowel function at 2 months, bowel bother at >1 year NS at other time points	>12
Cremers et al. (63)	SRT	3DCRT	EPIC	Function, 80	Function, 93	Function, 23	NR	Reference	NS	NA
Cortes-Gonzalez et al. (64)	SRT + NHT	3DCRT	QLQ-C30, PR-25	Symptoms, 24.7^b^	Symptoms, 9.4^b^	Symptoms, 50.4^b^	Function, 77.9^b^	Reference	NS	NA
Corbin et al. (65)	SRT/ART	IMRT	EPIC 26, IPSS	Irritations, 86 Incontinence, 78	Function, 89	Function, 36	NR	Baseline	NS	24
van Gysen et al. (66)	SRT/ART	IMRT	EPIC	Function, 82 Incontinence, 74	Function, 94	Function, 14	Physical component, 48	Baseline	NS	15
Berlin et al. (45)	SRT/ART	IG-IMRT	EPIC	Function, 85 Incontinence, 76	Function, 94 Bother, 90	Function, 25 Bother, 58	NR	Baseline	SS in favor of control arm: urinary irritation at 5 weeks, bowel function at 3 months, sexual function at 3 months NS at other time points	60

*QoL, quality of life; ART, adjuvant radiation therapy; RP, radical prostatectomy; SRT, salvage radiation therapy; NHT, neoadjuvant hormone therapy; 3DCRT, three-dimensional conformal radiation therapy; 4F, four field; IMRT, intensity-modulated radiation therapy; NA, not applicable; NR, not reported; NS, not significant; SS, statistically significant*.

*^a^Reference: reference values in age-matched healthy population. Baseline: patient’s pre-RT baseline*.

*^b^According to QLQ-C30 PR-25, higher mean for functional and general health domains shows higher functioning and lower mean for symptoms demonstrates less symptom burden*.

The impact of ART/SRT on sexual function represents a particular concern influenced by the low residual function post-prostatectomy. The latter also translates into challenges in evaluating the potential superimposed impact of ART/SRT on this QoL domain. Nonetheless, most of the studies that have evaluated this area have shown absence of ART/SRT impact on residual erectile function (see Table 2). This indeed contrasts with evidence of RT as primary treatment for localized disease, where a negative long-term impact has been reported (68). However, a possible explanation for this difference could be the prescribed doses between the two settings. Interestingly, an improvement trend of “sexual bother” subscale with time has been shown despite stability of sexual function scores, in keeping with patients getting used to a steady level of sexual functioning (45).

As an interesting corollary of these findings, the global quality of life was only transiently lower at 6 weeks in the SWOL QoL study, despite radiation toxicities and subsequent negative impact on GI- and GU-related QoL domains. In fact, it remained higher at 5 years for patients receiving ART as compared to control group (50). This in part could be explained by the effect of improved disease control on overall QoL in the RT arm. These latter observations serve to reinforce the complexity of QoL-related outcomes and studies. At any rate, considering the well-known mismatch in perception of QoL outcomes between physicians and patients (69), additional effort should be made by practitioners to convey unbiased information, which is more consistent with current evidence showing absence of detrimental effect (or even overall improvement) on QoL domains with the use of modern state-of-the-art post-prostatectomy RT.

## Conclusion and Future Steps

Postoperative adjuvant and salvage radiotherapy are effective and safe treatments in patients with high-risk factors or rising PSA after prostatectomy, respectively. Their underutilization might have several reasons, including concerns with overtreatment and radiation-related side effects. The current available data on toxicity demonstrate increased incidence of acute and long-term grade 2 events, but no significant increase of grade 3–4 long-term side effects with the use of ART/SRT. Although patients’ quality of life is affected transiently, it returns to pre-radiotherapy baseline during the first year after therapy. Despite the lack of randomized data comparing conventional with modern radiation techniques and lack of long-term follow-up of the latter, studies are consistent in suggesting an improved therapeutic index with the use of image-guided high-precision radiation, mainly due to better sparing of organs-at-risk translating into decreased toxicity rates. With the use of adjuvant radiation, a proportion of patients will be overtreated; however, present evidence does not seem robust enough to support similar effectiveness between delayed and adjuvant radiotherapy, and the latter should continue to represent the standard of care approach.

The literature on QoL and patient-reported outcomes after post-prostatectomy RT remains scarce, and continuous efforts in gathering prospective QoL data using validated tools seems necessary. Integration of QoL outcomes into both decision-making process and evaluation of treatments’ impact on survival outcomes remains an unmet challenge (70).

In conclusion, concerns with toxicities and/or impact in QoL outcomes should not preclude patients from gaining the proven benefits of either ART or SRT. Pending the results of prospective studies comparing adjuvant vs. early salvage radiotherapy, the former should represent the standard approach during shared decision-making process between physicians and patients for treatment individualization in men with localized prostate cancer after radical prostatectomy.

## Author Contributions

HR contributed in the literature search, analysis, and manuscript preparation. AB contributed in the literature search, analysis, and manuscript design and preparation.

## Conflict of Interest Statement

The authors declare that the research was conducted in the absence of any commercial or financial relationships that could be construed as a potential conflict of interest.
